# Managing COVID-19 infection in a Young Acute Myeloid Leukemia Patient Successfully With Antiviral and Granulocyte Colony Stimulating Factor: Playing on a Sticky Wicket

**DOI:** 10.7759/cureus.16589

**Published:** 2021-07-23

**Authors:** Dhruv Talwar, Sunil Kumar, Sourya Acharya, Shivam Khanna, Vidyashree Hulkoti

**Affiliations:** 1 Department of Medicine, Jawaharlal Nehru Medical College, Datta Meghe Institute of Medical Sciences, Wardha, IND

**Keywords:** covid19, acute myeloid leukemia, granulocyte colony stimulating factor, antiviral, steroid

## Abstract

The COVID-19 pandemic has drastically affected healthcare systems throughout the world. Though all domains of healthcare are busy battling this deadly pandemic, oncology care has taken a drastic hit due to cancer patients being immunocompromised and predisposed to acquire COVID-19 infection. Patients suffering from acute myeloid leukemia are at greater risk of acquiring Severe Acute Respiratory Syndrome Coronavirus 2(SARS CoV 2) infection along with developing complications related to COVID-19 due to the immunosuppression caused by the malignancy, as well as the high-intensity chemotherapy provided in acute myeloid leukemia. We report a case of 28-year-old male who was a known case of acute myeloid leukemia diagnosed three months ago, presented with high-grade fever with cough and breathlessness. Nasopharyngeal swab of the patient for SARS CoV2 by reverse transcriptase-polymerase chain reaction turned out to be positive. The patient was managed successfully with steroids, remdesavir, granulocyte colony-stimulating factor, and other supportive measures, and was discharged in a stable condition.

## Introduction

Ever since the beginning of the COVID19 pandemic there have been emerging challenges for healthcare professionals around the globe. Patients who are immunocompromised due to malignancies, HIV, chemotherapy, or history of a solid organ transplant have emerged as the weak ring of the lot with increased risk of contracting COVID-19 and increased risk of developing serious complications such as acute respiratory distress syndrome [1]. Due to the limitation of knowledge about SARS CoV2 and the absence of definitive treatment for the same, there has been great difficulty in battling the pandemic with a health care system that is already under an immense amount of pressure and with a scarcity of resources [2]. Acute myeloid leukemia has added to this burden of the healthcare system by puzzling the healthcare system and challenging the clinicians to draft a treatment strategy suitable for patients with acute myeloid leukemia as it is an immunocompromised state. The use of granulocyte colony-stimulating factors in COVID-19 has been established as a double-edged sword with evidence suggesting that granulocyte colony-stimulating factor leads to an increase in neutrophils in pneumonia as granulocyte colony stimulating factor has a major effect on myeloid compartment, thereby increasing proliferation, differentiation, survival and mobilization of neutrophils. This is associated with increased severity of COVID-19 on one hand, and on the other hand it can treat the lymphocytopenia of COVID-19 by increasing differentiation of macrophage [3]. Therefore, a clear risk-benefit ratio assessment should be carried out by the treating physician before giving granulocyte colony-stimulating factor in cases of COVID-19. We report a case of a 28-year-old male who was diagnosed three months ago as a case of acute myeloid leukemia and had received chemotherapy of 7+3 cycle of daunorubicin and cytarabine for the same. He presently complained of high-grade fever with cough and breathlessness and was tested positive for COVID-19 by a reverse transcriptase-polymerase chain reaction of the nasopharyngeal swab.

## Case presentation

**Figure 1:**
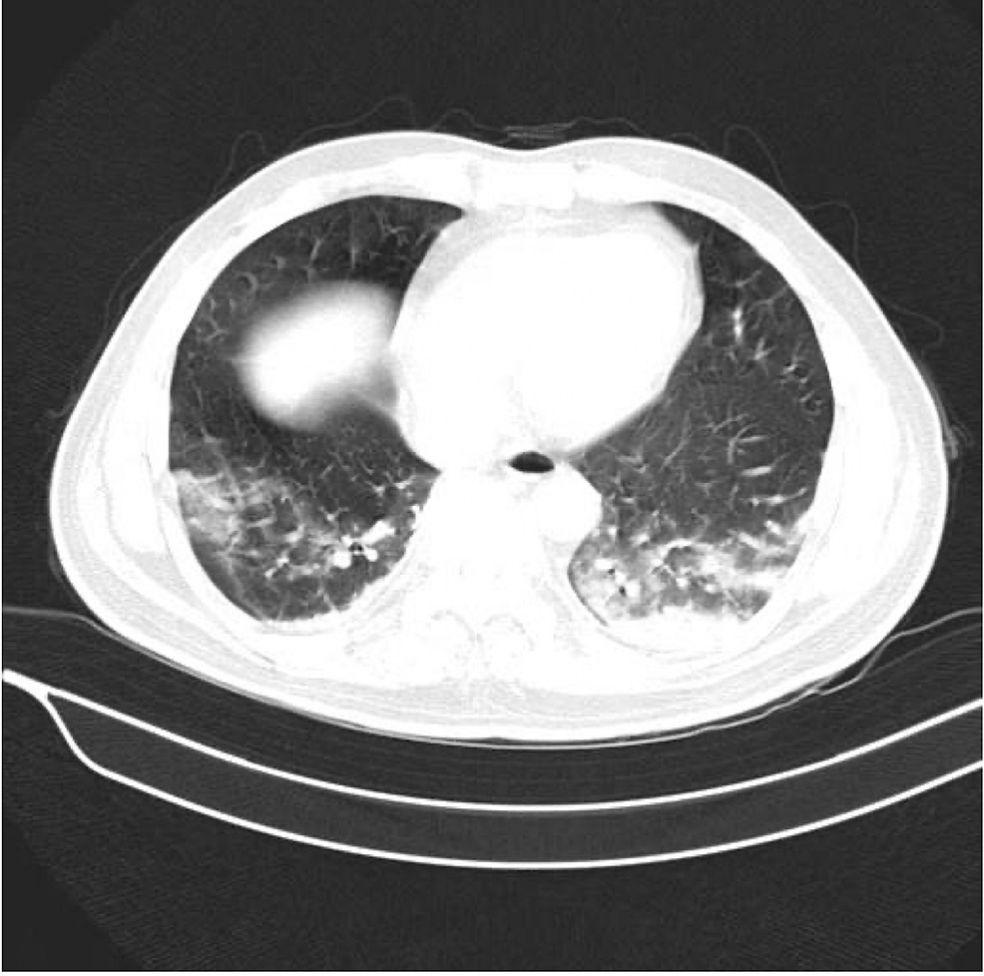
HRCT Thorax showing ground glass opacity suggestive of COVID-19

A 28-year-old male presented with the chief complaint of fever with chills along with cough and breathlessness for five days. He had a history of being diagnosed with acute myeloid leukemia three months ago, for which he had received chemotherapy cycle of 7+3 with daunorubicin and cytarabine. He had no history of hypertension, diabetes mellitus, tuberculosis or bronchial asthma. On general examination, the patient was febrile with a temperature of 101 °F, pulse of 106 beats per minute, blood pressure of 110/70 mm Hg in right arm supine position; pallor was present and SpO_2 _was 91 percent on room air. On systemic examination, trachea was centrally placed, there were fine crackles heard in the inframammary region of the chest, a systolic murmur was heard in the left second intercostal space which was functional, the abdomen was soft and non-tender with no hepatosplenomegaly and the patient was conscious and oriented. The patient was admitted for further evaluation and his nasopharyngeal swab for COVID-19 came positive by reverse transcriptase-polymerase chain reaction method. Blood investigations of the patient revealed pancytopenia and raised inflammatory markers with normal renal and liver function test (Table 1). HRCT chest was suggestive of bilateral lower lobe ground-glass opacities with an HRCT score of 5/25 and CORAD 6 (Figure 1). Patient was started on oxygen support along with remdesivir and steroids (Methylprednisolone 40mg twice Daily). He was given blood transfusion with two units of packed red cells transfusion in view of anemia. The patient was having persistent fever spikes along with lymphocytopenia in the blood picture. Hence, a careful risk-benefit assessment was made and granulocyte colony-stimulating factor was given to the patient for three days. Remdesivir was also given for 10 days. The patient improved clinically and oxygen was tapered. After 13 days of admission, the patient was ultimately discharged in stable condition with no fever spikes and an SpO2 of 95% on room air.

**Table 1:** Showing Lab investigations of the case

Lab Parameter	Result
Interleukin 6	69pg/ml (Normal range 5-15 pg/ml)
Serum Ferritin	661 ng/ml (Normal range 17.9-464 ng/ml)
Lactate Dehydrogenase)	719 u/ml (Normal Range 120-246 U/L)
CRP	59 mg/l (Normal Range 1-3 mg/l)
Hemoglobin	6.1 gm/dl (Normal range 13.8 -17.2 gm/dl)
MCV	80 fl (Normal range 80-100 fl)
Platelet Count	76000/mm3 (Normal range 150,000 to 450,000/mm3)
White Blood Cell Count	0.3 x 10^9^/L, Lymphocyte -10% (Normal Range 4.5 to 11.0 × 10^9^/L, Lymphocytes 20-40%)

## Discussion

**Figure 2:**
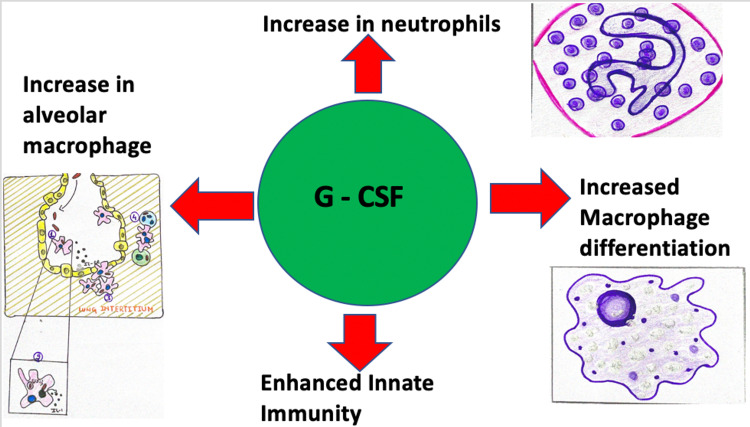
Showing effect of granulocyte colony stimulating factor on immunity

Acute myeloid leukemia is one of the commonest leukemia in India [4]. In acute myeloid leukemia, there is neoplastic proliferation of the white blood cells inside the marrow which may or may not involve the peripheral blood or non-hematopoietic tissues [5]. Acute myeloid leukemia is the malignancy of the myeloid lineage with myeloblast more than 20% in the bone marrow, loss of differentiation and pancytopenia. Hepatosplenomegaly and lymphadenopathy may or may not be present in a case of acute myeloid leukemia. Due to pancytopenia, the patient can be predisposed to contract various opportunistic infections. Such patients are also prone to contract COVID-19 infection. COVID-19 infection in a patient with acute myeloid leukemia is difficult to manage with dual-sided effects of granulocyte colony-stimulating factor. Granulocyte colony-stimulating factors can pronounce the neutrophilia in pneumonia, but it can also treat the lymphocytopenia of COVID-19 which is a marker of COVID’s severity [6]. Granulocyte colony-stimulating factor shortens the transit time for producing granulocytes from the bone marrow and also increases antigen-presenting cells. Granulocyte colony-stimulating factor has multiple clinical uses such as leukemia, graft vs host reaction and stem cell transplantation [7]. Alveolar macrophages act as primary defence against pathogens and granulocyte colony stimulating factor causes enhancement of alveolar macrophage along with increased innate immunity, which ultimately leads to protection against infections such as influenza (Figure 2). The increase in alveolar macrophages has been linked with immunity against influenza [8]. Our patient had severe lymphocytopenia, and a careful risk versus benefit ratio assessment was done and the patient was given granulocyte colony-stimulating factor. Granulocyte colony stimulating factor lead to an increase in lymphocyte counts and total white blood cell count from 300 white blood cells (10% lymphocyte) to 1100 white blood cells (30% lymphocytes). It was expected that with the leukocytopenia and lymphocytopenia, our patient would have developed severe complications of COVID-19. However, timely administration of granulocyte colony stimulating factor might have helped in treating COVID-19 successfully with no major complications. Other measures which were taken to prevent COVID-19 related complications and to prevent severe COVID infection were that our patient was also continued on remdesivir for a period of ten days considering his immunocompromised status and he was isolated in a separate room for his treatment in order to prevent opportunistic infections. Absence of development of complications or any increase in clinical symptoms in our immunocompromised patient points towards the synergistic benefit of granulocyte colony stimulating factor along with extended antiviral administration with steroids. COVID-19 should be kept in the differentials in cases of acute myeloid leukemia who contract fever irrespective of the presence of absence of typical symptoms. Our case report emphasizes the importance of individual tailoring of prescriptions in COVID-19 with special cases. In the absence of definitive treatment, there are conflicts and confusion in deciding the appropriate treatment protocol for patients suffering from malignancies and hence, special care should be given while treating such patients. Growth factor support in our patient helped in early recovery and discharge, hence preventing mortality in our case.

## Conclusions

A detailed understanding of granulocyte colony stimulating factor and leukemia is essential in tackling a patient with acute myeloid leukemia with COVID-19. A thorough assessment of risk versus benefit ratio may help the treating physician in choosing to give granulocyte colony stimulating factor which increases alveolar macrophages and innate immunity along with increasing lymphocytes, thus helping in preventing morbidity and mortality.
